# Assessment of autonomic function in patient with COVID-19 and other infectious diseases using a wearable smart band connected to a mobile application

**DOI:** 10.3389/fpsyt.2025.1618004

**Published:** 2026-02-03

**Authors:** Eun Bit Bae, Jang Wook Sohn, Jeong Yeon Kim, Kyu-Man Han

**Affiliations:** 1 Korea University Research Institute for Medical Bigdata Science, Korea University College of Medicine, Republic of Korea; 2 Division of Infectious Diseases, Department of Internal Medicine, Korea University College of Medicine, Republic of Korea; 3 Department of Psychiatry, Korea University College of Medicine, Republic of Korea

**Keywords:** coronavirus disease (COVID)-19, heart rate variability (HRV), isolation, monitoring, post-pandemic, smart band, stress, wearable device

## Abstract

The negative impact of the coronavirus disease 2019 (COVID-19) pandemic on mental health, including that of movement restrictions that unintentionally contributed to its deterioration, has been widely reported. However, the effects of isolation and related factors remain unclear. To explore the physiological, psychological, and lifestyle factors that affected stress levels in individuals with confirmed COVID-19 undergoing isolation, we used a modified version of a commercially available wearable device for the purpose of real-time monitoring. The study included 60 infection patients affected by infectious diseases (30 with confirmed COVID-19 undergoing isolation at home, and 30 inpatients at our institution with other infectious diseases). Based on the data distribution, we conducted correlation analyses within each group and evaluated the relationship between variables using conservative methods, general linear regression, and linear mixed models. The groups comparison was evaluated using an independent-samples t-test. Stress scores in the study population showed significant associations with psychological and lifestyle factors, but not with psychiatric scale scores. According to the linear model, caffeine consumption affected the root mean square of successive differences (RMSSD) (p = 0.031). In participants with confirmed COVID-19 undergoing isolation at home, alcohol consumption and anxiety levels showed strong correlations with RMSSD (p< 0.005), although this was not evident in linear models. Stress scores were significantly higher in participants with COVID-19, whereas RMSSD deviation from the mean of an age-matched Korean cohort was significantly lower than that in patients with other infectious diseases. This study suggests that while perceived stress may influence parasympathetic function in all patients with infectious diseases, this effect may be particularly pronounced in those with COVID-19 undergoing isolation. These individuals are more likely to experience stress and anxiety, and their parasympathetic function may be compromised (reflected in a reduction of heart rate variability). Our results suggests that lifestyle factors and perceived stress influences parasympathetic function in under stressful conditions associated with confinement, and that these factors should be considered in the management of individuals with COVID-19 in isolation.

## Introduction

Coronavirus disease 2019 (COVID-19) is at present transitioning into an endemic phase, with daily life largely returning to normality, with mask mandates and restrictions imposed during the acute phase no longer applied. However, circumstances have differed markedly from the present situation over the past 5 years. In 2020, as COVID-19 infection with severe acute respiratory syndrome coronavirus 2 (SARS-CoV-2) cases surged worldwide, governments implemented strict public health measures, including restrictions on public activity and lockdowns of public services (1, 2). Numerous studies have examined the devastating impact of the COVID-19 pandemic on global mental health (3), revealing significant effects on depression and anxiety levels (4), sleep disturbances (5) and neuropsychiatric effects (6, 7). Considering the period encompassed by the study, these differences may be attributed to prolonged exposure to pandemic-related restrictions (8). Similarly, the World Health Organization reported a global increase of 25% in the prevalence of depression and anxiety during the pandemic (9). Other comparative studies have also documented a significant decline in mental health before and during the COVID-19 pandemic (10). A review in *Nature Medicine* further highlighted that loneliness and depressive symptoms increased during the initial phase of the pandemic and remained elevated up until early 2021 in healthy individuals (3). Notably, severe psychiatric symptoms can lead to suicidal behavior and ideation (11).

However, no previous studies have specifically investigated functional changes in the autonomic system during the isolation period. Two studies have examined heart rate variability (HRV) and physiological changes in relation to the pandemic. Bourdillon et al. (8) analyzed heart rate and HRV changes in healthy individuals before, during, and after public lockdowns, reporting a significant increase in heart rate during the quarantine period compared with pre-lockdown levels. Additionally, the root mean square of successive differences (RMSSD) of 80% healthy participants showed a significant decrease during and after quarantine compared with pre-quarantine levels (7). Another study by Ong et al. investigated heart rate and sleep duration variability across 20 countries during lockdowns in Oura Ring users (12). Both studies suggested that lockdowns may have influenced autonomic function. However, physiological changes related to isolation in the post-COVID-19 period remain largely unexplored.

During the COVID-19 pandemic, digital healthcare technologies integrating wearable Internet of Things (IoT) devices and mobile applications have been extensively developed and incorporated into daily healthcare practice. A widely used function of smartwatches and smart bands is stress measurement, estimated by evaluating the function of the autonomic nervous system. Various types of physical and psychological stress can decrease parasympathetic autonomic function, which is reflected in reduced HRV values. For instance, several articles have reported that some patients with affective disorders, such as depression (13, 14) and anxiety (15, 16), exhibit lower resting-state HRV, reflecting reduced parasympathetic activity compared with that of healthy controls. Despite these pieces of evidence, there are still controversies regarding the relationship between HRV, psychiatric disorders, and psychiatric scales, as other studies failed to observe a significant relationship between RMSSD and psychiatric disorders (17–20). This suggests that additional research on this subject is warranted.

In the post-pandemic era, digital healthcare technology may be instrumental in the monitoring of individuals during quarantine periods, helping to prevent long-term adverse health outcomes and ensuring continued access to physical and mental healthcare services. This technology helps to mitigate health disruptions in the general population. More broadly, maintaining healthcare resilience during quarantine periods by guaranteeing access of vulnerable populations to the healthcare system is essential for preventing the exacerbation of health disparities (21, 22).

RMSSD and the standard deviation of N-N intervals (SDNN) are recognized as key metrics related to HRV that reflect parasympathetic nervous system activity, and are normally recorded by smart bands (23). Previous research has demonstrated that RMSSD can be reliably estimated from ultra-short-term recordings (10–60 s) (24). Alali has previously reported that RMSSD showed a significant and high correlation (r > 0.7) with gold standard of 5-minute ECG derived RMSSD and more reliable in ultra-short-term recordings (25, 26), whereas the SDNN measurement required longer period (26). Moreover, in contrast with SDNN, RMSSD is less affected by longer-term heart rate changes such as those caused by the circadian rhythm (27, 28).

Multiple factors and stressors influencing RMSSD have been identified (29), but it is unclear which social and environmental factors are relevant in the case of individuals in isolation. This study assessed i) parasympathetic function (estimated by HRV and RMSSD) in individuals with confirmed COVID-19 subjected to isolation and in inpatients affected by any infectious disease; ii) relationship between parasympathetic function, lifestyle, and mental health; and iii) differences using the individual’s real-time physiological RMSSD deviation from Korean cohort norm data between confirmed COVID-19 isolation patients and those with other infections patients.

## Methods

### Participants and study design

**Figure 1:**
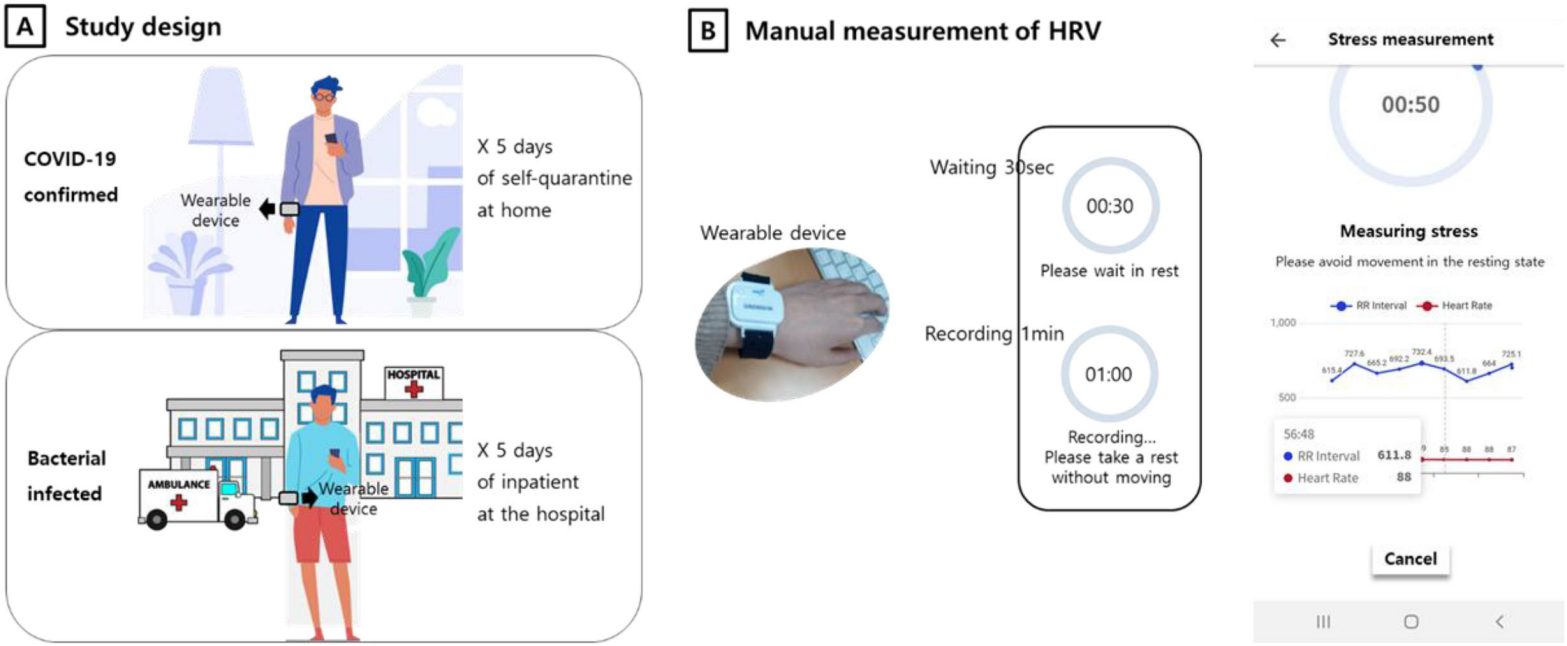
Post-COVID-19 monitoring system based on smart Internet of Things devices. **(A)** Participants with confirmed COVID-19 are self-isolated at home, whereas inpatients with other infections participated in the clinical trial at the hospital. **(B)** Modified wearable device developed by AmoSense (Korea) and detail of the mobile application display showing pulse-to-pulse intervals recorded during manual measurements. When the measurements were performed automatically, the data did not appear in the mobile application display.

Considering the importance of healthcare during isolation, a specialized clinical trial was prospectively designed as an observational study to evaluate parasympathetic autonomic function in individuals with confirmed COVID-19 and in inpatients with other infections (**Figure 1A**). Considering that fever is a common symptom of infection, participants were allocated to two infection groups: those with COVID-19 and inpatients admitted to the Division of Infectious Diseases, Department of Internal Medicine of our institution, without a COVID-19 diagnosis.

In addition, as part of a national research project, a commercial smart band (Amoband, AmoSense Co., Ltd; Cheonan-si; Republic of Korea) was modified to be used in combination with a mobile application and monitor physiological variables in participants under mandatory isolation at home and in hospitalized patients diagnosed with infectious diseases other than COVID-19, such as urinary tract infections, pneumonia, and etc. (Supplementary Information, **Supplementary Table S1**).

Another part of the research project involved the development by the application company Softnet Co., Inc (Seoul, Republic of Korea) of a specialized mobile application to monitor and report on physiological health status as well as on lifestyle factors associated with mental health in isolated individuals. HRV was recorded using a modified version of the Amoband.

We included participants who provided written informed consent to participate in the clinical trial. Written consent was obtained from both participants and their legal guardians in the case of underage participants. The exclusion criteria were as follows: i) unstable cardiovascular conditions that could influence HRV, including implanted cardiac devices or the use of cardiovascular medication (27); ii) limited ability to use a wearable device, such as intolerance due to skin irritation on the wrist; and iii) difficulty completing the study requirements, including unwillingness to wear the device during the 5-d study period.

Participants were enrolled from two institutions (**Figure 1A**): Thirty cases with confirmed COVID-19 were recruited from the Yeongcheon Public Health Center in Yeongcheon City, Gyeongsangbuk-do, South Korea, whereas 30 patients diagnosed with bacterial infections (e.g., urinary infection or pneumonia, SI **Supplementary Table S1**) were recruited from the Korea University Anam Hospital. We presumed that all participants might have a fever. We did not limit medication use in a general procedure for participants. All participants received treatment according to a general procedure.

During the initial visit, participants were informed of the clinical trial procedure and asked to provide written informed consent to participate. On the same day, each participant was sent a text message containing a link that allowed them to install an application compatible with the developed wearable device.

The clinical trial period spanned 5 consecutive working d, from Monday to Friday. During the trial period, participants with confirmed COVID-19 were mandated to self-isolate for approximately 1 week. The remaining participants were inpatients admitted for over 1 week to the general wards of the Division of Infectious Diseases, Department of Internal Medicine of our institution (**Figure 1A**). All participants were instructed to wear the device continuously for 24 h, allowing for a maximum of 2 h without wearing it.

All procedures were performed in accordance with the principles of the Declaration of Helsinki. The Institutional Review Board of Korea University Anam Hospital (No. 2022AN0568) approved the clinical trial, and participants were recruited from December 2022 to July 2023.

### RMSSD measurement and data processing

This study used data derived from photoplethysmography (PPG) to calculate pulse rate variability. Previously, a couple of studies reported a high correlation between ultra-short-term of PPG-derived PRV and electrocardiography outcomes in 5-min recording (30, 31). We therefore used RMSSD values provided by the mobile application for the study, which were derived from PPG readings obtained by the sensor (**Figure 1B**).

The commercially available device Amoband was modified by its manufacturer (Amosense Co., Ltd) as part of a research project focused on developing monitoring systems for individuals in isolation. The PPG sensor included in the device is designed to detect pulse signals. The largest pulse wave recorded was used to calculate the pulse-to-pulse interval (inter-beat interval) (32, 33).

Detected signals were digitized by the wearable device, and the resulting data was transferred to the mobile application through a Bluetooth connection for the calculation of RMSSD (**Figure 1B**). RMSSD was selected as the primary HRV parameter to monitor autonomic nervous system function based on its widespread use in commercially available smart bands.

The RMSSD value was derived from the pulse-to-pulse intervals by calculating continuous pulse waves using the following formula (25, 34, 35):


$$RMSSD=\sqrt{\frac{1}{N-1}\left[\sum_{i=1}^{N-1}\left(PPI_{i+2}-PPI_{i+1}\right)-\left(PPI_{i+1}-PPI_{i}\right)\right]}\;$$

RMSSD values were obtained through both manual and automatic measurements. For manual measurements, participants activated the “stress measurement” function within the application by tapping a designated button. After a 30-s waiting period to ensure data stability and accuracy, heart rate and pulse-to-pulse intervals were recorded in real-time through the mobile application during 1 min. Following the recording, the application calculated the corresponding RMSSD values (**Figure 1B**). For automatic measurement, HRV was recorded at hourly intervals, with the recording sessions having the same duration as in the case of manual measurement (1 min). Heart rate and RMSSD values were immediately available from the application upon recording completion.

Since an internal pilot study revealed instability in the automatic measurements, resulting in a high failure rate in the recording of HRV values, manual and automatic measurements were used in combination during the study period.

### Lifestyle information

Autonomic function is directly influenced by caffeine, alcohol, and nicotine consumption (36). To investigate factors affecting isolation, participants were asked four questions regarding substance use and exercise activities: “How many cups of coffee do you consume in a week?,” “How much/many times do you smoke in a day?,” “How many times do you consume alcohol in a week?,” and “How many times do you exercise in a week?.” Data on caffeine, alcohol, and nicotine consumption as well as exercise patterns were collected from the participants when they first initiated the application after installation. The responses were assumed to reflect usual consumption and activity levels, rather than temporary levels immediately before HRV measurement.

### Psychological and psychiatric scales

The mental health of participants was assessed using two types of scales: visual analog scales (VAS) ranging from 0 to 10, and internationally validated psychiatric scales (37, 38). Subjective mental stress was assessed using four VAS scales (39, 40): VAS stress, depression, anxiety, and sleep quality. These scales allowed participants to self-report their perceived mood and condition. To cross-validate the subjective VAS measurements, internationally standardized psychiatric scales, including the Patient Health Questionnaire (9-item, PHQ-9), Generalized Anxiety Disorder scale (7-item, GAD-7), and Insomnia Severity Index (ISI), were used to assess depression, anxiety, and insomnia, respectively (41–44). All scales were manually accessed at the discretion of the participant, and they were encouraged to respond to both types of scales. Validated Korean versions of all scales were used.

### Statistical analysis

During manual stress measurement, the R-R interval was calculated in real time and stored in the database. However, the number of manually recorded stress measurements was insufficient to estimate the corresponding R-R intervals, as data collection was dependent on participant discretion. Therefore, only RMSSD values, which were calculated and reported in real time via the application, were used in the analysis. The raw RMSSD data collected through automatic and manual measurements were pre-processed to remove undetected signals reflected as null values.

To minimize the effects of age and sex, which are known to affect HRV (36), we used a South Korean normative RMSSD data matched for age and sex for the purpose of comparison. To compare the two groups of participants in our study with healthy individuals, the differences in RMSSD were calculated as follows:

*Differences in RMSSD* = Participant RMSSD – Average RMSSD from Korean cohort (SI **Supplementary Table S2**) (45).

RMSSD data distribution was assessed with the Shapiro-Wilk test, and the Mann-Whitney U test was used to compare the RMSSD, considering distribution. Four types of analysis were conducted in the following order: i) Separate correlation analysis in each group due to differences in RMSSD data distribution (Pearson correlation for the confirmed COVID-19 cases and Spearman correlation for the inpatients). ii) General linear regression conducted to assess linear association between RMSSD values, substance use information, and psychiatric scales in the entire study population and within the confirmed COVID-19 group. iii) A linear mixed effects model used to estimate both fixed effects and random effects of observations in the total participants, with mean RMSSD used to examine associations with lifestyle factors and psychological and psychiatric scales. iv) A comparison analysis conducted using an independent-sample t-test. Leven’s equality variance test showed that the inpatient group did not exhibit equal variance (p < 0.05). Data distribution and linear mixed model were analyzed via Python version 3.12. The statistical analysis was performed using IBM SPSS Statistics version 27 (IBM Corp., Armonk, NY, USA).

## Results

The descriptive statistics for all participants and the number of the participants included in the study are displayed in **Table 1**, with additional information shown in **Supplementary Table S1** (SI).

**Table 1:** Descriptive data for all participants.

Total participants	Average	SD	Min	Max	Number of participants
Group	30:30				60
Age	41.56	20.31	15	82	60
Sex	11:49				
Avg RMSSD	30.80	4.00	23.21	34.79	48 (1,748)
VAS stress	2.31	2.31	0	10	46 (97)
Regression	Average	SD	Min	Max	Number of participants
Age	38.76	18.83	15.0	82.0	46
Sex	8:38				46
Avg RMSSD	53.71	8.83	34.4	84.0	46
SD RMSSD	14.57	4.17	3.1	22.4	44
Min RMSSD	24.67	17.20	1.0	84.0	46
Max RMSSD	82.57	17.41	49.0	110.0	46
No RMSSD	37.93	33.12	1.0	116.0	46
Coffee	0.67	0.90	0.0	3.0	46
Smoking	0.67	2.68	0.0	15.0	46
Exercise	0.35	0.82	0.0	3.0	46
Alcohol	0.48	1.03	0.0	4.0	46
PHQ-9	4.20	3.85	0.0	11.0	10
GAD-7	1.20	1.23	0.0	4.0	10
ISI	4.30	3.02	1.0	11.0	10
VAS Stress	2.16	2.06	0.0	7.0	35
VAS Depression	1.28	1.88	0.0	7.0	27
VAS Anxiety	1.20	1.74	0.0	6.0	27
VAS Insomnia	1.11	1.66	0.0	6.0	27

Avg, average; SD, standard deviation; RMSSD, root mean square successive differences; VAS, visual analogue scale; PHQ-9, Patient Health Questionnaire-9; GAD-7, Generalized Anxiety Disorder scale-7; ISI, Insomnia Severity Index.

RMSSD validity results were as follows: The artifact values and rates were 861/2,423 for confirmed COVID-19 cases in isolation [35.5%] and 276/1,879 for inpatients [14.4%]). The total number of RMSSD readings analyzed was 918 for confirmed COVID-19 cases and 830 for inpatients. RMSSD values were distributed normally in the confirmed COVID-19 cases, but significantly deviated from a normal distribution in inpatients (Shapiro–Wilk test: confirmed COVID-19 cases, W = 0.997, p-value = 0.059; inpatients, W = 0.990, p-value = 0.000). Considering the RMSSD distribution, the Mann–Whitney U test was used to compare RMSSD values and no significant difference (t = -0.858, p = 0.391) was identified between the two groups (COVID-19 M = 52.6, SD = 16.43; Other = 53.3, SD = 16.92).

Significant linear correlations were observed in the study population between the VAS outcomes for stress and for depression, anxiety, and insomnia (**Table 2**). In addition, the VAS outcomes for stress demonstrated significant associations in the linear regression analysis with four lifestyle factors (**Table 2**): Coffee consumption (F[6] = 6.68, p< 0.001), smoking (F[6] = 6.85, p< 0.001), alcohol consumption (F[7] = 4.90, p = 0.001), and exercise (F[6] = 6.57, p< 0.001). In contrast, psychiatric scales, including the PHQ-9, GAD-7, and ISI, did not exhibit any significant linear associations with either VAS outcomes for stress or mean RMSSD.

**Table 2:** General linear model results for all participants.

Outcomes	Fixed predictor	Df	MS	F	p	Corrected R^2^
SD RMSSD	AvgVAS stress	21	16.11	0.55	0.889	-0.426
	Coffee	3	17.29	0.99	0.406	0.000
	Smoking	3	27.24	1.64	0.196	0.042
	Alcohol	4	37.70	2.46	0.061	0.120
	Exercise	3	32.44	1.99	0.130	0.065
Avg RMSSD	Avg VAS stress	22	75.14	0.71	0.768	-0.234
	PHQ-9	7	54.04	2.22	0.345	0.488
	GAD-7	5	72.61	4.55	0.084	0.663
	ISI	6	65.96	6.36	0.079	0.781
**VAS stress**	**VAS depression**	12	6.53	9.20	< 0.001^*^	**0.797**
	**VAS Anxiety**	14	5.84	10.92	< 0.001^*^	**0.847**
	**VAS Insomnia**	10	7.74	11.32	< 0.001^*^	**0.805**
	GAD-7	5	4.36	2.21	0.274	0.430
	ISI	7	3.79	3.21	0.406	0.660
	**Coffee**	6	14.20	6.68	< 0.001^*^	**0.500**
	**Smoking**	6	14.35	6.85	< 0.001^*^	**0.508**
	**Alcohol**	7	11.57	4.90	0.001^*^	**0.445**
	**Exercise**	6	14.11	6.57	< 0.001^*^	**0.496**

Df, degree of freedom; p, p-value; Avg, Average; VAS, visual analog scale; PHQ-9, Patient Health Questionnaire-9; GAD-7, Generalized Anxiety Disorder-7; ISI, Insomnia Severity Index; Avg RMSSD, average root mean square of the successive differences; SD RMSSD, standard deviation of RMSSD; VAS stress, mean VAS stress; Bonferroni corrected significance level (*αBonf =* 0.00278, ^*^p<0.002). All significant results were presented in bold font.

As a more conservative method compared to correlation, we applied a linear mixed effects model. The results showed an association between mean RMSSD and coffee consumption (p = 0.031), and between VAS outcomes for stress and smoking (p = 0.013). However, these associations were no longer significant after applying the Bonferroni correction (**Table 3**). Age was the only significant predictor for VAS outcomes for stress.

**Table 3:** Linear mixed model results for all participants. (n = 35).

Outcomes	Fixed predictor	Coeff.	SE	Z	p	LL	UL
Avg RMSSD	Intercept	61.148	4.353	14.046	< 0.001	52.616	69.681
	Sex	0.523	4.534	0.115	0.908	-8.365	9.410
	Age	-0.109	0.085	-1.279	0.201	-.275	.058
	Avg VAS Stress	-1.032	0.690	-1.497	0.134	-2.384	.319
	Coffee	-3.990	1.845	-2.162	0.031	-7.607	-.373
	Smoking	-0.186	0.654	-0.285	0.776	-1.467	1.095
	Exercise	-0.904	2.525	-0.358	0.720	-5.853	4.045
	Alcohol	3.582	2.409	1.487	0.137	-1.140	8.304
VAS Stress	Intercept	3.745	.463	8.091	< 0.001	2.838	4.652
	Sex	-0.990	.823	-1.204	0.229	-2.602	.622
	Age	-0.042	.015	-2.738	0.006^*^	-.073	-.012
	Coffee	0.008	.348	0.022	0.983	-.675	.690
	Smoking	0.263	.106	2.476	0.013	.055	.471
	Exercise	0.230	.445	0.517	0.605	-.642	1.101
	Alcohol	-0.261	.439	-0.595	0.552	-1.122	.600

Coeff., coefficient; SE, standard error; z, z-score; p, p-value; 95% confidence interval level, LL, Lower limit (*α* = 0.025*)*; UL, upper limit (*α* = 0.975*)*; Avg, Average; VAS, visual analog scale; RMSSD, root mean square of the successive differences; Bonferroni corrected significance level (*αBonf =* 0.007; Mean VAS Stress, *αBonf =* 0.008, ^*^p< 0.008).

Due to differences in data distribution, correlation analysis was conducted separately for each group (**Table 4**, SI **Supplementary Table S3**). Significant associations between age and coffee consumption (p< 0.001), and between alcohol consumption and standard deviation (SD) of RMSSD (p = 0.003) were found in confirmed COVID-19 cases. GAD-7 scores were significantly associated with the mean and maximum RMSSD values (both p = 0.003), and VAS outcomes for stress were significantly associated with outcomes for depression, anxiety, and insomnia (p< 0.007). However, inpatients showed significant associations between mean RMSSD values and VAS outcomes for stress (P = 0.010), and between alcohol consumption and exercise habits (P< 0.001, **Supplementary Table S4**).

**Table 4:** Pearson correlation results for COVID-19 confirmed participants.

COVID-19	1	2	3	4	5	6	7	8	9	10	11	12	13	14	15	16
1.Age	0															
2. AV RMSSD	0.609	0														
3.SD RMSSD	0.956	0.135	0													
4. Min RMSSD	0.449	0.019	0.197	0												
5. Max RMSSD	0.129	0.011	0.000*	0.539	0											
6. VAS Stress	0.111	0.972	0.335	0.797	0.552	0										
7. Coffee	0.000*	0.447	0.409	0.615	0.636	0.037	0									
8. Smoking	0.660	0.731	0.059	0.232	0.221	0.073	0.863	0								
9. Exercise	0.369	0.537	0.155	0.591	0.336	0.109	0.210	0.332	0							
10. Alcohol	0.935	0.374	0.003*	0.063	0.183	0.635	0.060	0.045	0.052	0						
11. PHQ	0.167	0.818	0.254	0.434	0.678	0.166	0.553	0.452	0.310	0.856	0					
12. GAD	0.752	0.003*	0.205	0.614	0.003*	0.216	0.942	0.017	0.488	0.682	0.493	0				
13. ISI	0.068	0.908	0.737	0.858	0.407	0.062	0.118	0.663	0.221	0.439	0.018	0.361	0			
14. VAS dep	0.916	0.605	0.815	0.536	0.262	0.005*	0.880	–	0.532	0.602	0.812	0.274	0.287	0		
15. VAS anx	0.475	0.320	0.990	0.209	0.217	0.006*	0.471	–	0.873	0.730	0.836	0.263	0.359	0.000*	0	
16. VAS insom	0.725	0.645	0.790	0.407	0.247	0.002*	0.805	–	0.761	0.533	0.846	0.254	0.431	0.000*	0.000*	0

1. Age; 2. Average RMSSD; 3. Standard deviation RMSSD; 4. Minimum RMSSD; 5. Max RMSSD; 6. Visual analogue scale Stress; 7. Coffee; 8. Smoking; 9. Exercise; 10. Alcohol; 11. Patient Health Questionnaire-9; 12. General Anxiety Disorder-7; 13. Korean version of the Insomnia Severity Index; 14. VAS depression; 15. VAS anxiety; 16. VAS insomnia. All questionnaires were provided in a validated Korean version. Significance level (*p < 0.05)

Similarly, a linear association was observed between the SD of RMSSD and alcohol consumption within the confirmed COVID-19 cases (**Table 5**, F[4] = 3.63, p = 0.023) that disappeared after applying the Bonferroni correction.

**Table 5:** General linear model results for participants with confirmed COVID-19.

Factors		df	MS	F	p	Corrected R^2^
SD RMSSD	Avg VAS stress	15	18.10	0.61	0.783	-0.442
	Coffee	3	13.85	0.71	0.556	-0.039
	Smoking	4	19.80	1.07	0.398	0.012
	Alcohol	4	46.58	3.63	0.023	0.314
	Exercise	4	22.29	1.24	0.327	0.040
Avg RMSSD	Mean VAS stress	15	64.05	0.52	0.844	-0.618
	GAD-7	4	83.88	3.95	0.144	0.628
	ISI	6	66.16	29.00	0.141	0.960
Avg VAS stress	VAS depression	9	2.71	1.53	0.560	0.324
	VAS Anxiety	8	2.94	2.23	0.346	0.497
	VAS Insomnia	7	3.05	1.91	0.321	0.389

Df, degree of freedom; p, p-value; SD, standard deviation; RMSSD, root mean square of the successive differences; Avg, Average; VAS, visual analogue scale; GAD-7, Generalized Anxiety Disorder-7; ISI, Insomnia Severity Index; Bonferroni corrected significance level (*αBonf =* 0.00455).

**Figure 2:**
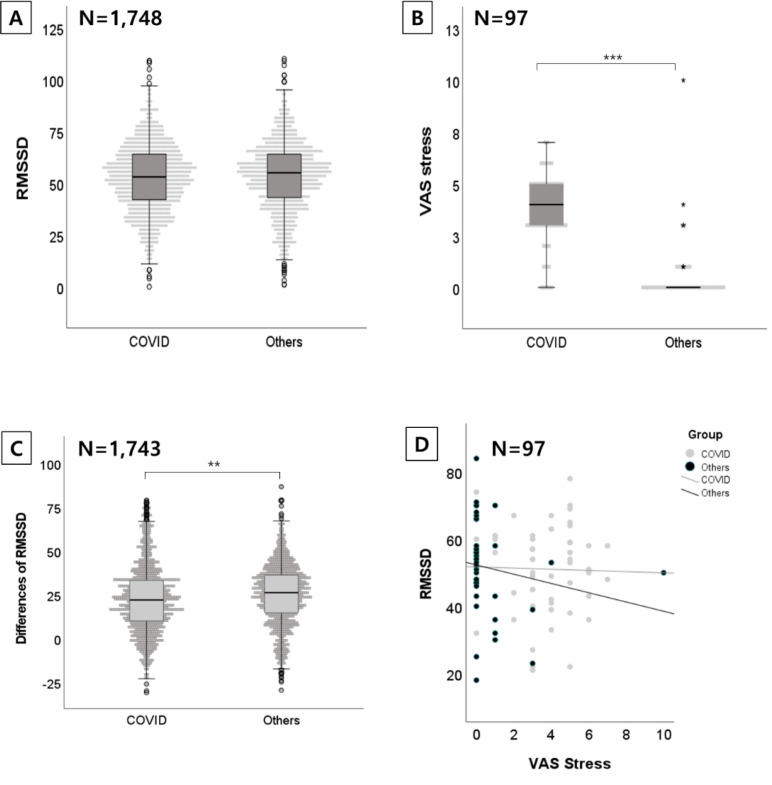
Results of the comparison between participants with confirmed COVID-19 in isolation and inpatients with other infection. **(A)** RMSSD distribution in participants with COVID-19 and with other infectious diseases (N = 1,748). **(B)** Comparison of VAS outcomes for stress between participants with COVID-19 and with other infectious diseases (N = 97). **(C)** Differences in RMSSD, calculated as the difference between the RMSSD from each participant and average RMSSD from the age-matched Korean cohort. **(D)** Correlation between RMSSD and VAS outcomes for stress in participants with COVID-19 and with other infectious diseases (N = 97).

The RMSSD distribution did not show significant differences between groups (p = 0.391). A comparative analysis of the RMSSD differences with an age- and sex-matched cohort, confirmed COVID-19 cases (M = 3.6, SD = 1.81) exhibited significantly higher stress levels compared to that of inpatients (M = 0.62, SD = 1.74; t (90.1) = 8.2; p< 0.001; **Figure 2B**). However, the results indicated no significant differences in RMSSD values between the two groups (t [1746] = -0.858, p = 0.391; **Figure 2A**). This aligned with the correlation results.

These findings suggest that psychological stress is elevated in home isolation patients, despite no significant differences in autonomic function parameters between the two groups.

The differences between individual RMSSD values and the age-matched average RMSSD from the Korean cohort were significantly higher in the inpatient group (M = 24.21, SD = 17.66) than in confirmed COVID-19 cases (M = 22.86, SD = 19.19; t [1740.7] = -2.65, p = 0.008; **Figure 2C**, **Table 6**, **Supplementary Table S5**). However, notably, VAS outcomes for stress were significantly higher in the confirmed COVID-19 group (M = 3.60, SD = 1.811) compared to that of inpatients (M = 0.62, SD = 1.738, t [90.132] = 8.217, p< 0.001; **Figures 2A–C**). Although there were no significant differences between the groups for raw RMSSD data, the deviation from the mean RMSSD of the age-matched Korean cohort was significantly different in the confirmed COVID-19 cases and in inpatients. Thus, autonomic function in confirmed COVID-19 cases was more closely aligned than that of inpatients with the averages of the Korean cohort.

**Table 6:** Group comparison results between patients with COVID-19 and those with other infectious diseases.

	t	df	p	Diff. *M*	Diff. *SE*	LLCI	ULCI
RMSSD	-0.858	1746	0.391	-0.685	0.798	-2.250	0.880
**VAS Stress**	8.217	90.132	< 0.001^*^	2.981	0.363	2.260	3.702
RMSSD	-0.674	1741	0.500	-0.537	0.797	-2.099	1.025
**Differences with norm**	-2.646	1740.731	0.008^*^	-2.34	0.883	-4.067	-0.605

t, t-score; df, degree of freedom; p, p-value; Diff.*M*, Mean difference (COVID-19 – Others); Diff.*SE*, Standard Error differences (COVID-19 – Others); LLCI, Low limit of credential interval; ULCI, Upper limit of credential interval. RMSSD differences, Differences with norm, RMSSD differences between participants, and age/sex matched cohort norm. Group mean and standard deviation were displayed in SI **Supplementary Table S4**. VAS Stress and Differences with norm did not show equal variances in the Levene’s test. If variances were not significantly different, df could be 95 for VAS Stress, and 1,741 for Differences with norm. Bonferroni corrected significance level (*αBonf =* 0.025). All significant results were presented in bold font.

## Discussion

This study investigated the effectiveness of an IoT-based smart band, used in combination with a mobile application. In this study, individuals diagnosed with COVID-19 and undergoing home isolation, as well as inpatients with other infections, were evaluated using real-time HRV monitoring. We assumed that the inpatients environment might closely resemble the conditions of the isolation group. HRV, environmental factors, and mental health parameters were comprehensively compared between isolation individuals and inpatients. Our findings revealed several novel considerations. Individuals subjected to isolation exhibited noteworthy associations between RMSSD, anxiety, and alcohol consumption, and between stress levels and other psychiatric conditions such as depression, anxiety, and insomnia (**Table 4**). Accordingly, the results of the age- and sex-matched comparison showed significantly higher perceived stress levels and smaller RMSSD deviations from the comparison cohort average in confirmed COVID-19 cases undergoing isolation.

Previously, RMSSD has shown significant negative associations with psychological stress measured by VAS (8, 46–48) Consistent with these findings, higher scores on psychiatric scales such as the PHQ-9, GAD-7, and ISI (used to measure depression, anxiety, and insomnia, respectively) have been negatively associated with RMSSD (49–51). In addition, recent studies that made use of wearable devices have demonstrated significant associations between the increasing RMSSD and decreasing scores on the PHQ-9, GAD-7, and ISI scales (52, 53).

The results for psychiatric scales (PHQ-9, GAD-7, and ISI) did not align with those previously reported. Two possible explanations for the inconsistent results between the VAS and psychiatric scales were considered. First, the smaller number of responses for the psychiatric scales compared to those for the VAS scales may have negatively affected the results (N = 10, **Table 2**). Second, the psychiatric scales assess mental health during the previous 2 weeks, whereas VAS scales capture real-time or daily self-reported status, potentially contributing to differences in findings.

Anxiety and insomnia exhibited linear correlations with the average RMSSD (GAD-7, R^2^ = 0.663; ISI, R^2^ = 0.781), but these associations were not significant in the entire study population (GAD-7, p = 0.084; ISI, p = 0.079; **Table 2**). This may be due to the low statistical power derived from a small sample size. Regarding the impact of substance use on physical stress, significant reductions in RMSSD have been reported among current smokers and alcohol consumers (54–56), whereas caffeine intake and physical activity have been associated with significant increases in heart rate and RMSSD (57, 58). The linear model in this study aligns with previous findings, particularly in the confirmed cases of COVID-19 (F[4] = 3.63, p = 0.023; **Table 3**).

Our results highlighted that VAS outcomes for stress and coffee consumption may influence the mean RMSSD in all patients. For the confirmed COVID-19 group that underwent isolation, two key factors in particular may influence parasympathetic regulation: perceived stress and psychiatric conditions associated with isolation status as well as alcohol consumption. The group comparison results revealed novel findings: the RMSSD deviation from the average of the age-matched cohort was significantly lower in confirmed COVID-19- cases, which is consistent with their much higher stress levels according to the VAS. However, the RMSSD distribution did not show significant differences. Similarly, previous studies have reported significantly lower values for RMSSD in individuals with COVID-19 compared to that of healthy controls (8, 59). COVID-19 causes cardiovascular symptoms, and therefore, isolation status lead to a highly stressful situation (demonstrated by the enhanced VAS outcomes for stress and strong association between them and the outcomes from psychiatric scales), contributing to decreased cardiovascular function reflected in reduced values for RMSSD (60, 61).

Since this study was conducted during the post-pandemic and endemic periods of COVID-19, a high prevalence of prior infection with SARS-CoV-2 in the inpatient group was assumed, which may explain why the average RMSSD did not show significant differences between groups.

To summarize, this study found that COVID-19 remains a stressful condition, even in the post- and endemic periods, compared with other infectious diseases. Our findings indicated that these stressful conditions may result in lower RMSSD compared to that in individuals affected by other infectious diseases. These results highlight the need for targeted mental health interventions and continuous monitoring of isolation conditions, including lifestyle factors during post-COVID-19 care. These insights, which associate perceived stress, lifestyle, psychiatric symptoms, and autonomic function, provide a foundation for mitigating the long-term effects of the pandemic on both mental and physical health.

### Limitations

The contradictory results of the regression between VAS outcomes for stress and those for the psychiatric scales may have been caused by the small sample size, as only 10 participants completed the psychiatric scales, of which, 6–8 of them were included in regression analysis. Despite this limitation, perceived stress is strongly associated with various factors, including psychiatric scales and physiological stress related to substance use habits.

Regarding the HRV estimate, RMSSD was automatically calculated through the mobile application. Therefore, the R-R interval was not stored in a database, preventing the derivation of additional HRV indices, such as the SD of NN intervals, low-frequency, and high-frequency. Therefore, we included the SD of RMSSD in the statistical analyses to assess RMSSD stability. The lifestyle factors were assessed as habitual weekly frequencies rather than time-locked daily exposures. Consequently, we could not analyze acute physiological responses to specific events (e.g., alcohol consumption on a given day), limiting temporal causal inference regarding daily variability. Future studies should employ ecological momentary assessment (EMA) to capture day-level dynamics. A limitation in group comparisons is that the normative data were obtained from heterogeneous environments, recording a 5−minute ECG, and it is longer than 1 minute of PPG.

Further studies are recommended to evaluate the influences of other physiological and medical characteristics. Despite the limitations inherent to the use of wearable technologies, this study may open new possibilities for large-scale and longitudinal research with user-friendly, wearable devices.

## Conclusion

This study enhances our understanding of the effects of isolation on stress and HRV in individuals that have gone through the acute phase of COVID-19 through the innovative use of IoT smart band technology for real-time monitoring. The incorporation of smart wristbands into post-COVID-19 research represents a significant step forward in the objective and continuous monitoring of physiological responses associated with stress and autonomic regulation. The findings provide encouraging evidence that digital health tools yield meaningful insight into the long-term psychophysiological consequences of isolation and contribute to the development of prevention and rehabilitation strategies. Moreover, commercially available devices open new possibilities for large-scale and longitudinal research. This study highlights the stress experienced by quarantined individuals and provides practical insight for the development of targeted mental health interventions and policy formulations. Future research should validate these results at the individual level and focus on a broader sample population to enhance generalizability.

## Data Availability

The raw data supporting the conclusions of this article will be made available by the authors, without undue reservation.
