# Supplementary material for: Assessment of autonomic function in patient with COVID-19 and other infectious diseases using a wearable smart band connected to a mobile application
Source: Front Psychiatry. 2026 Feb 3;16:1618004. doi: 10.3389/fpsyt.2025.1618004 (PMC12960646; doi:10.3389/fpsyt.2025.1618004)
Supplement: Supplementary file 1 [file Table1.docx]

Frontiers in Psychiatry Supplementary Information

**Assessment of Autonomic Function in Patient with COVID-19 and Other Infectious Diseases Using a Wearable Smart Band Connected to a Mobile Application**

Eun Bit Bae^1*^, Jang Wook Sohn^1,2*^, Jeong Yeon Kim^2^, Kyu-Man Han^3^

^1^ Korea University Research Institute for Medical Bigdata Science, Korea University College of Medicine, Republic of Korea

^2^ Division of Infectious Diseases, Department of Internal Medicine, Korea University College of Medicine, Republic of Korea

^3^ Department of Psychiatry, Korea University College of Medicine, Republic of Korea

Table S1. Inpatients’ information.

| **Subject ID** | **Age** | **Gender** | **Diagnosis** |
| --- | --- | --- | --- |
| S31 | 67 | M | Infectious spondylitis |
| S32 | 72 | F | Adrenal insufficiency |
| S33 | 82 | M | Acute pyelonephritis |
| S34 | 61 | M | Infective endocarditis |
| S35 | 67 | M | Acute pyelonephritis |
| S36 | 35 | F | Acute pyelonephritis |
| S37 | 68 | M | Acute prostatitis |
| S38 | 64 | F | Acute pyelonephritis |
| S39 | 45 | M | Chest wall abscess |
| S40 | 24 | F | Dengue |
| S41 | 69 | F | Urinary tract infection |
| S42 | 42 | F | Infective endocarditis |
| S43 | 19 | F | Acute colitis |
| S44 | 52 | M | Complicated hepatic cyst |
| S45 | 37 | M | Acute pyelonephritis |
| S46 | 28 | F | Cutaneous Leishmaniasis |
| S47 | 82 | F | Urinary tract infection |
| S48 | 61 | M | Urinary tract infection |
| S49 | 32 | M | Urinary tract infection |
| S50 | 62 | M | Urinary tract infection |
| S51 | 67 | M | Pneumonia |
| S52 | 82 | F | Urinary tract infection |
| S53 | 70 | F | Infectious spondylitis |
| S54 | 68 | M | Pneumonia |
| S55 | 68 | M | Colitis |
| S56 | 22 | F | Pneumonia |
| S57 | 56 | F | Acute pyelonephritis |
| S58 | 31 | F | Liver abscess |
| S59 | 49 | F | Liver abscess |
| S60 | 61 | F | Cutaneous abscess of buttock |

Table S2. Descriptive data for the Korean cohort used for comparison (previously reported by Kim & Woo, 2011)

|  | RMSSD description in age group (year) | | | | | | | | | F | P-value | |
| --- | --- | --- | --- | --- | --- | --- | --- | --- | --- | --- | --- | --- |
| Age / | ≤ 29 | | | 30 - 39 | | 40 - 49 | | 50 ≤ | |  |  |  |
| Gender | Mean | SD | | Mean | SD | Mean | SD | Mean | SD |  |  |  |
| Men | 36.10 | 19.18 | | 31.82 | 16.73 | 28.22 | 17.89 | 23.21 | 16.66 | 30.23 | < 0.001 | |
| N = | 168 | | | 804 | | 1,181 | | 518 | | 2,671 | | |
| Women | 33.88 | | 15.57 | 34.79 | 19.24 | 30.42 | 18.19 | 27.70 | 27.07 | 4.19 | | 0.006 |
| N= | 183 | | | 222 | | 202 | | 105 | | 712 | | |

RMSSD, root mean square of successive differences; SD, Standard deviation

Citation: Kim GM, Woo JM. Determinants for heart rate variability in a normal Korean population. J Korean Med Sci. 2011;26(10):1293-8. doi: 10.3346/jkms.2011.26.10.1293.

Table S3. Results for the SD RMSSD dependent variable in the linear mixed model from all participants.

|  | **Coeff.** | **SE** | **z** | **p** | **LL** | **UL** |
| --- | --- | --- | --- | --- | --- | --- |
| Intercept | 16.716 | 1.523 | 10.976 | .000 | 13.731 | 19.701 |
| Gender | .393 | 2.269 | .173 | .862 | -4.054 | 4.840 |
| Age | -.015 | .036 | -.429 | .668 | -.085 | .054 |
| Mean VAS Stress | -.412 | .266 | -1.546 | .122 | -.934 | .110 |
| Coffee | .079 | .914 | .087 | .931 | -1.712 | 1.870 |
| Smoking | -.162 | .285 | -.567 | .571 | -.721 | .397 |
| Exercise | .013 | 1.181 | .011 | .991 | -2.302 | 2.327 |
| Alcohol | -1.505 | 1.076 | -1.399 | .162 | -3.614 | .604 |

SD, standard deviation; RMSSD, root mean square of the successive differences; Coeff., coefficient; SE, standard error; z, z-score; p, p-value; 95% confidence interval level, LL, Lower limit (*α* = 0.025*)*; UL, upper limit (*α* = 0.975*)*; Avg, Average; VAS, visual analog scale

Table S4. Spearman correlation results for other infection group.

|  | 1 | 2 | 3 | 4 | 5 | 6 | 7 | 8 | 9 | 10 | 11 | 12 | 13 |
| --- | --- | --- | --- | --- | --- | --- | --- | --- | --- | --- | --- | --- | --- |
| 1.Age | 0 | 0.141 | 0.918 | 0.574 | 0.184 | 0.344 | 0.815 | 0.430 | 0.471 | 0.471 | 0.523 | 0.221 | 0.055 |
| 2.Sex no. | 0.141 | 0 | 0.399 | 0.937 | 0.498 | 0.781 | 0.202 | 0.574 | 0.210 | 0.210 | 0.336 | 0.363 | 0.287 |
| 3.AV RMSSD | 0.918 | 0.399 | 0 | 0.658 | 0.576 | 0.103 | 0.010* | 0.105 | 0.523 | 0.523 | 0.688 | 0.856 | 0.833 |
| 4.SD RMSSD | 0.574 | 0.937 | 0.658 | 0 | 0.000* | 0.003* | 0.197 | 0.745 | 0.376 | 0.376 | 0.835 | 0.401 | 0.452 |
| 5.Min RMSSD | 0.184 | 0.498 | 0.576 | 0.000* | 0 | 0.006* | 0.862 | 0.966 | 0.285 | 0.285 | 0.970 | 0.405 | 0.547 |
| 6.Max RMSSD | 0.344 | 0.781 | 0.103 | 0.003* | 0.006* | 0 | 0.101 | 0.517 | 0.101 | 0.101 | 0.523 | 0.969 | 0.951 |
| 7.VAS Stress | 0.815 | 0.202 | **0.010*** | 0.197 | 0.862 | 0.101 | 0 | 0.165 | 0.908 | 0.908 | 0.677 | 0.546 | 0.183 |
| 8.Coffee | 0.430 | 0.574 | 0.105 | 0.745 | 0.966 | 0.517 | 0.165 | 0 | 0.012 | 0.012 | 0.318 | 0.442 | 0.031 |
| 9.Exercise | 0.471 | 0.210 | 0.523 | 0.376 | 0.285 | 0.101 | 0.908 | 0.012 | 0 | 0.000* | 0.156 | 0.323 | 0.084 |
| 10.Alcohol | 0.471 | 0.210 | 0.523 | 0.376 | 0.285 | 0.101 | 0.908 | 0.012 | 0.000* | 0 | 0.156 | 0.323 | 0.084 |
| 11.VAS dep | 0.523 | 0.336 | 0.688 | 0.835 | 0.970 | 0.523 | 0.677 | 0.318 | 0.156 | 0.156 | 0 | 0.426 | 0.026 |
| 12.VAS anx | 0.221 | 0.363 | 0.856 | 0.401 | 0.405 | 0.969 | 0.546 | 0.442 | 0.323 | 0.323 | 0.426 | 0 | 0.408 |
| 13.VAS insom | 0.055 | 0.287 | 0.833 | 0.452 | 0.547 | 0.951 | 0.183 | 0.031 | 0.084 | 0.084 | 0.026 | 0.408 | 0 |

1. Age; 2. Sex no.; 3. Average RMSSD; 4. Standard deviation RMSSD; 5. Minimum RMSSD; 6. Max RMSSD; 7. Visual analogue scale Stress; 8. Coffee; 9. Exercise; 10. Alcohol; 11. VAS depression; 12. VAS anxiety; 13. VAS insomnia. All questionnaires were provided in a validated Korean version.

Table S5. Group average and standard deviation of RMSSD, VAS stress, and RMSSD differences with Korean cohort norm.

|  | Group | N | Average | Standard deviation | t | p-value |
| --- | --- | --- | --- | --- | --- | --- |
| RMSSD | COVID-19 | 918 | 52.61 | 16.426 | -.858 | .391 |
|  | Others | 830 | 53.29 | 16.918 |  |  |
| **VAS Stress** | COVID-19 | 55 | 3.60 | 1.811 | 8.217 | **<.001^*^** |
|  | Others | 42 | 0.62 | 1.738 |  |  |
| RMSSD | COVID-19 | 918 | 52.75 | 16.321 | -.674 | .500 |
|  | Others | 830 | 53.29 | 16.918 |  |  |
| **RMSSD Differences with cohort norm** | COVID-19 | 918 | 22.88 | 19.19 | -2.646 | **.008^*^** |
|  | Others | 830 | 25.21 | 17.66 |  |  |

RMSSD, root mean square of the successive differences; VAS Stress, Visual Analogue Scale of Stress; cohort norm, Korean cohort norm (Table S2).
